# Disrupting the Oncogenic Synergism between Nucleolin and Ras Results in Cell Growth Inhibition and Cell Death

**DOI:** 10.1371/journal.pone.0075269

**Published:** 2013-09-24

**Authors:** Sari Schokoroy, Dolly Juster, Yoel Kloog, Ronit Pinkas-Kramarski

**Affiliations:** Department of Neurobiology, Tel-Aviv University, Ramat-Aviv, Israel; Enzo Life Sciences, Inc., United States of America

## Abstract

**Background:**

The ErbB receptors, Ras proteins and nucleolin are major contributors to malignant transformation. The pleiotropic protein nucleolin can bind to both Ras protein and ErbB receptors. Previously, we have demonstrated a crosstalk between Ras, nucleolin and the ErbB1 receptor. Activated Ras facilitates nucleolin interaction with ErbB1 and stabilizes ErbB1 levels. The three oncogenes synergistically facilitate anchorage independent growth and tumor growth in nude mice.

**Methodology/Principal Findings:**

In the present study we used several cancer cell lines. The effect of Ras and nucleolin inhibition was determined using cell growth, cell death and cell motility assays. Protein expression was determined by immunohistochemistry. We found that inhibition of Ras and nucleolin reduces tumor cell growth, enhances cell death and inhibits anchorage independent growth. Our results reveal that the combined treatment affects Ras and nucleolin levels and localization. Our study also indicates that Salirasib (FTS, Ras inhibitor) reduces cell motility, which is not affected by the nucleolin inhibitor.

**Conclusions/Significance:**

These results suggest that targeting both nucleolin and Ras may represent an additional avenue for inhibiting cancers driven by these oncogenes.

## Introduction

The Ras family of small GTPases transmits extracellular signals, which are initiated by cell-surface receptors and serve to regulate various cellular processes including cell growth, differentiation, motility and cell death [1]. Signals transmitted by activated Ras induce activation of multiple effectors [1,2]. Ras signaling is activated in a large number of human cancers [3]. Mutations of codons 12, 13 and 61 in *Ras* result in constitutively active Ras, and activating mutations of the three major Ras isoforms (H, K and N) have been found in more than 33% of human cancers [4-6].

Since Ras signaling represents a junction for many extracellular signals, Ras and its effectors are targets for therapeutic intervention. Ras is post-translationally modified by the addition of a farnesyl lipid group that allows its attachment to the cell membrane. S-trans*, trans-*farnesylthiosalicylic acid (FTS; also known as Salirasib) is a synthetic Ras inhibitor that structurally resembles the carboxy-terminal farnesylcysteine group common to all Ras proteins. FTS acts as an effective Ras antagonist in cells, affecting Ras interaction with the membrane, dislodging Ras from its anchorage domains and facilitating its degradation, thereby reducing cellular Ras levels [7,8]. FTS inhibits the growth of H-Ras-, K-Ras- and N-Ras-transformed rodent fibroblasts [9,10]. In addition, FTS can inhibit the anchorage-independent growth of LNCaP, PC-3 and CWR-R1 cells [11,12]. Moreover, several tumor cells do not undergo apoptosis when treated with FTS. These include pancreatic [13], colon [14] and lung cancer cell lines that express mutant K-Ras [15].

Nucleolin is a ubiquitously expressed acidic phosphoprotein with key functions in transcription, and in the synthesis and maturation of ribosomes [16,17]. It is involved in important aspects of cell proliferation and cell growth [18,19]. Nucleolin is localized primarily to the nucleoli, but it undergoes nuclear-cytoplasmic shuttling and is also found on the cell surface of some types of cells [17,20,21]. Inhibition of cell-surface nucleolin and nucleolin activities suppresses growth of breast, prostate and glioma cell lines, which also express high levels/ or activated Ras protein [16,22-24]. We recently identified non-nucleolar nucleolin as an ErbB receptor-interacting protein [25]. This interaction leads to receptor dimerization and activation as well as to increased colony growth in soft agar [25]. The ErbB1 nuclear localization sequence (NLS) is important for nucleolin-ErbB1 interaction, dimerization and activation [26]. In addition, more recently, we have identified a crosstalk between nucleolin, ErbB1 and Ras proteins [27]. Importantly, we have demonstrated that expression of activated H-Ras (G12V), nucleolin and ErbB1 enhance cell transformation as evident by increased colony formation in soft agar and increased tumor volume in nude mice.

These results were the driving force to conduct the present study. In this *in-vitro* study, we examined the impact of FTS and GroA (AS1411) treatment on cell growth of various human cancer cell lines, and determined the contribution of the combined treatment to cell viability, cell motility and anchorage independent growth. Our results demonstrated that FTS and GroA combined treatment affects Ras and nucleolin intracellular localization, inhibits cell growth, induces cell death, reduces cell motility and inhibits anchorage independent growth.

## Materials and Methods

### Materials and buffers

Salirasib (FTS, S-trans*, trans*-farnesylthiosalicylic acid) was purchased from Concordia Pharmaceuticals. For FTS preparation, FTS powder was washed in chloroform, the solution was then vaporized by liquid nitrogen twice. The resulted powder was dissolved in 0.1% DMSO in medium supplemented with 10% FBS to concentration of 100 mM. The aptamer GroA (GROA/AS1411) and the inactive oligomer Cro, were purchased from IDT (Jerusalem, Israel) as unmodified desalted oligonucleotides as previously described [16,28]. The oligonucleotides were reconstituted in DDW to 1 mM concentration and incubated at 65°C for 15 minutes. Methylene blue (1% in boric acid); Propidium iodide (0.5mg/ml in DDW); Thiazolyl blue tetrazolium bromide (MTT, 5mg/ml in PBS); were purchased from Sigma. Agar Noble (2%, 0.6% in DDW); BD Becton Dickinson. Staurosporine (STS) was purchased from Sigma. Stock solution 500 µM diluted for final concentration of 200 nM. The Caspase inhibitor Q-VD(OMe)-OPh was purchased from R&D systems, diluted in 0.1% DMSO for stock solution of 10 mM. Antibodies were obtained from the following sources: monoclonal mouse antiActin (MP biomedicals, CA; 691001), monoclonal mouse anti pan Ras (Calbiochem, OP 40), polyclonal rabbit anti Casapase 3 (Cell signaling, 9662), polyclonal rabbit anti Nucleolin (C23, Santa cruz, sc-13057).

### Cell cultures

The human colon cancer cells DLD-1 were grown in RPMI-1640 (Gibco), human colon cancer HCT-116 cell line were grown in Mccoy’s 5A (Sigma), prostate cancer cells PC-3 were grown RPMI-1640 (Gibco), prostate cancer cells DU-145 were grown in Dulbecco's modified Eagle’s DMEM (Biological Industries), MDCK cells were grown in Dulbecco's modified Eagle’s DMEM (Biological Industries), Rat-1 fibroblast cells and H-Ras-transformed Rat-1 cells (EJ cells) were grown in DMEM. All media were supplemented with antibiotics and 10% heat-inactivated fetal bovine serum (FBS; Hyclone, Thermo Scientific). Cells were incubated at 37°C in 5% CO_2_ in air, and the medium was changed every 3-4 days. One day before treatment the cells were plated at ~50% confluence in medium supplemented with 5% fetal bovine serum (10% for EJ and Rat-1 cells). Treatments with FTS, with or without GroA, were according to the indicated concentration (cells are treated with 0.1% DMSO as a control for FTS and Cro as a control for GroA) for the times specified in each experiment. The human cell lines, and MDCK cells were from ATCC. The Rat-1 and Rat-1-EJ cells were a gift from Prof. Y Yarden (Weizmann Institute) [10].

### Assays of cell survival and cell death

Cells were plated in medium supplemented with 5% FBS (10% for EJ and Rat-1 cells) and treated as indicated for the different experiments. Cell numbers were determined by the methylene blue assay. For this purpose, the cells were fixed with 4% formaldehyde in phosphate-buffered saline for 2 hours, then washed once with 0.1 M boric acid (pH 8.5) and incubated with the DNA-binding dye methylene blue (1% in boric acid) for 20 minutes at room temperature. The cells were then washed three times and lysed with 0.1 M HCl. Absorbance was measured with a Tecan Spectrafluor Plus spectrophotometer (Mannedorf, Switzerland) at 595 nm. Cell viability was calculated as the ratio of absorbance in treated cultures to that in untreated control cultures. Nuclear staining and nuclear morphology scored dead cells. To estimate the number of dying cells, live cells were incubated for 10 minutes with 1 μg/ml of the fluorescent DNA dye bisbenzimide (Hoechst 33258; Sigma). After staining, the cells were photographed with an Olympus motorized inverted research microscope Model IX81 (20× magnification). The percentage of dead cells was estimated by calculating the number of Hoechst-stained nuclei relative to the total cell number in each field.

### Cell cycle analysis

Cells were plated in medium supplemented with 5% FBS and treated as indicated. Following treatments, the cells were trypsinized, washed once with PBS, and fixed in cold methanol for 15 minutes. Fixed cells were washed once with PBS and incubated at 4°C for 30 minutes. RNase A (0.05 mg/ml) and propidium iodide (0.05 mg/ml) were added and the stained cells were analyzed in a fluorescence-activated cell sorter (FACScan; Becton Dickinson, Franklin Lakes, NJ) within 30 minutes. Percentages of cells at different stages of the cell cycle were determined using the WinMDI 2.9 software.

### Anchorage independent assay

Noble agars (2% and 0.6%) were prepared in double-distilled water and autoclaved. The 2% agar was melted and mixed with medium (concentrated X^2^ with 20% FBS), and the mixture (50 µl) was placed in 96-well plates to provide the base agar (at a final concentration of 1%). The cells were suspended in medium (concentrated X^2^ mixed with 0.6% agar), and 50 µl of the mixture were plated on the base agar. 100 µl of medium (×1, 10% FBS) containing the indicated treatments (concentrated×2) was added to the wells. The plates were incubated for 7-14 days at 37°C. Colonies were then stained with 25 µl 3-(4,5-dimethylthiazol-2-yl)-2,5-diphenyltetrazolium bromide (5 mg/ml) and photomicrographed. The number of colonies per well (>0.01 mm^2^) was determined using the ImagePro software.

### Scratch-induced migration assay

Cells were plated in six-well plates. A day after treatment, by the time confluency was reached, a scratch wound was inflicted at each well and the resulting gap was imaged at the indicated time points. The area of the scratch was quantified by using the ImageJ software (arbitrary units).

### Microscopy

For microscopy analysis of Ras and nucleolin localization, we used MDCK cells stably expressing GFP-K-Ras and RFP-Nucleolin, which were previously described [27] and DLD-1 cells. Cells were seeded on coverslip coated with poly-L-Lysine in DMEM supplemented with 5% FBS for MDCK or RPMI supplemented with 5% FBS for DLD-1. The cells were then stimulated with the indicated treatments and fixed with 4% paraformaldehyde in PBS. The cells were later examined under a fluorescence microscope at 63×magnification with the Zeiss 510 META confocal microscope. For double staining of endogenous Ras and Nucleolin in DLD-1 cells, fixed cells were immunostained with rabbit anti-nucleolin antibodies (1:50) and mouse anti- Pan Ras (1:150) followed by Alexa Fluor 488-labeled goat anti-mouse and Alexa Fluor 546- labeled goat anti-rabbit secondary antibodies (1:500; Invitrogen). Cells were examined by fluorescence microscopy at 63×magnification with Zeiss 510 META confocal microscope.

### Lysates preparation and immunoblott analysis

After the indicated treatment, cells were lysed in solubilization buffer (50 mM HEPES pH 7.5, 150 mM NaCl, 10% glycerol, 1% Triton X-100, 1 mM EDTA pH 8, 1 mM EGTA pH 8, 1.5 mM MgCl2, 200 μM Na3VO4, 150 nM aprotinin, 1 μM leupeptin and 500 μM 4-(2-aminoethyl) benzenesulfonyl fluoride hydrochloride (Sigma). Lysates were cleared by centrifugation and a boiling sample buffer was added. Lysates were resolved by sodium dodecyl sulfate polyacrylamide gel electrophoresis through 10%–12.5% polyacrylamide gels, and were electrophoretically transferred to nitrocellulose membranes. Membranes were blocked for 1 hour in TBST buffer (0.05 M Tris-HCl pH 7.5, 0.15 M NaCl, and 0.1% Tween 20) containing 6% milk, and then blotted with primary antibodies for 2 hours. Secondary antibody linked to horseradish peroxidase was then added for 1 hour. Immunoreactive bands were detected with the enhanced chemiluminescence reagent.

### Statistical analysis

All experiments were performed at least 3 times unless otherwise stated. Experimental differences were tested for statistical significance using Student’s t-test. P-value of <0.05 was considered as significant.

## Results

### Nucleolin and Ras inhibition affect cell growth

**Figure 1:**
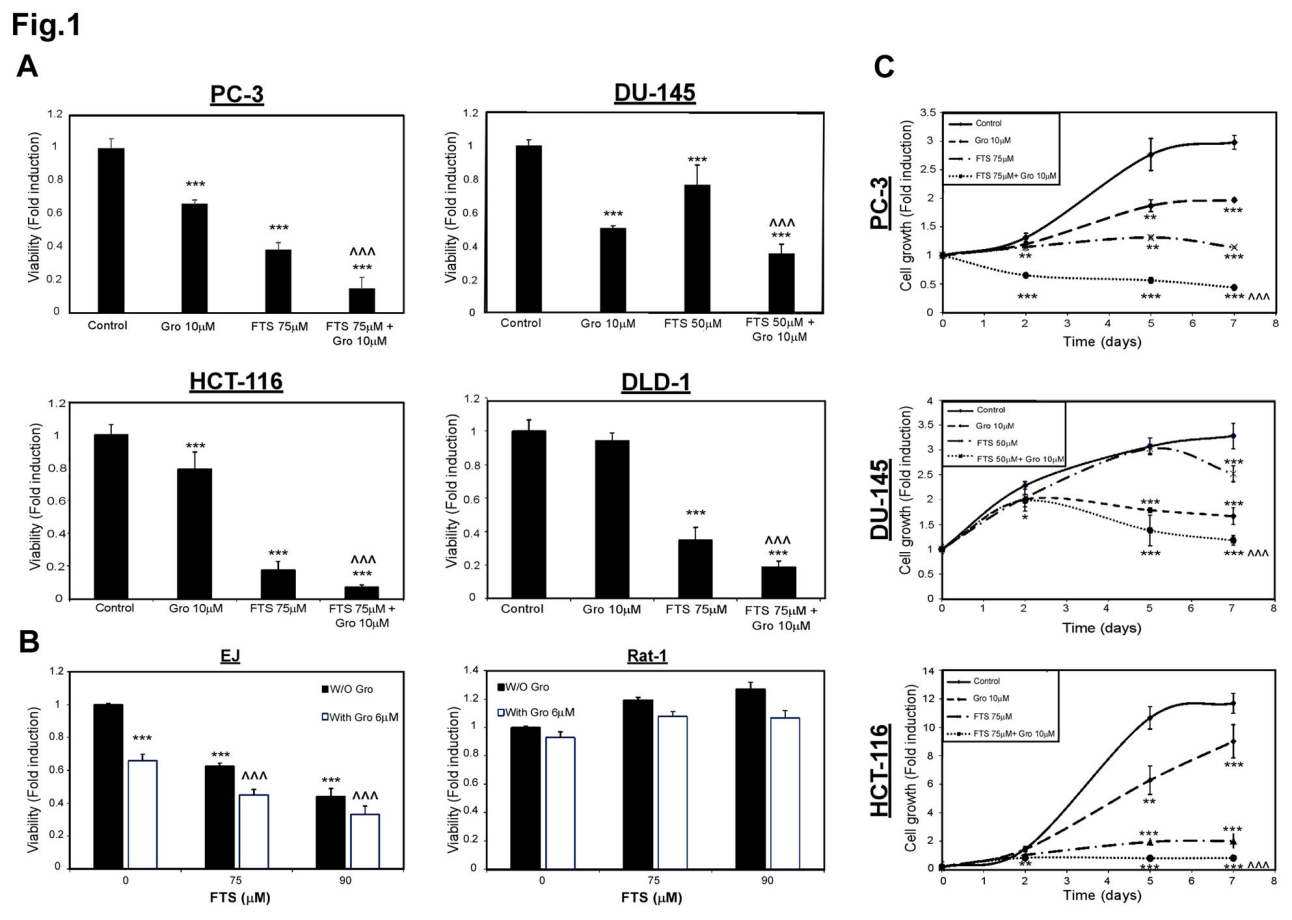
GroA enhances the inhibitory effect of FTS on cell viability and cell growth in a time dependent manner. (A) PC-3, DU-145, HCT-116 and DLD-1 cells were treated for 7 days with 50 or 75 µM FTS, in the presence or absence of 10 µM GroA. (B) EJ and Rat-1 cells were treated for 5 days with 75 μM or 90 μM FTS, in the presence or absence of 6 µM GroA. The cells were then tested for cell viability using the methylene blue staining assay. (C) PC-3, DU-145 and HCT-116 cells were treated with 50 or 75 µM FTS, in the presence or absence of 10 µM GroA. Methylene blue assay was performed 0, 2, 5 and 7 days later. The Results are presented as fold induction of the control and are presented as the mean ± S.D (**, p < 0.05 ; ***, p < 0.005, each treatment compared to the control ; ^ ^ ^, p< 0.005, combination compared to each treatment alone). These experiments were repeated at least three times with similar results.

In order to test the effect of nucleolin and Ras inhibition on tumor cell growth, we used two inhibitors: FTS (salirasib) [29-31], a powerful Ras inhibitor, and GroA (AS1411), an aptamer that targets cell surface nucleolin [16,32-34]. First, we examined the ability of Ras inhibition by FTS and nucleolin inhibition by the aptamer GroA to reduce the number of cells (Figure 1A). As shown, in two prostate cancer cell lines (PC-3 and DU-145) and in two colon cancer cell lines (HCT-116 and DLD-1), FTS and GroA inhibited cell growth, while the combined treatment was significantly more effective than each of the treatments alone. We next performed time course analysis to estimate the degree of cell growth inhibition at various time periods following treatment. As shown in Figure 1C, in all the cell lines examined, each of the treatments inhibited cell growth. However, the combined treatment was significantly more effective in inhibition of cell growth. The observed cell growth inhibition started on day 2 and lasted at least 7 days (Figure 1C). We also compared the effect of the treatment on naïve Rat1 fibroblasts and on Ras transformed Rat-1 (EJ) fibroblasts. As shown in Figure 1B, the drugs were significantly effective in inhibition of EJ cell growth. However, the treatment barely affected the naïve Rat1 cells.

### The combined treatment induces cell death

**Figure 2:**
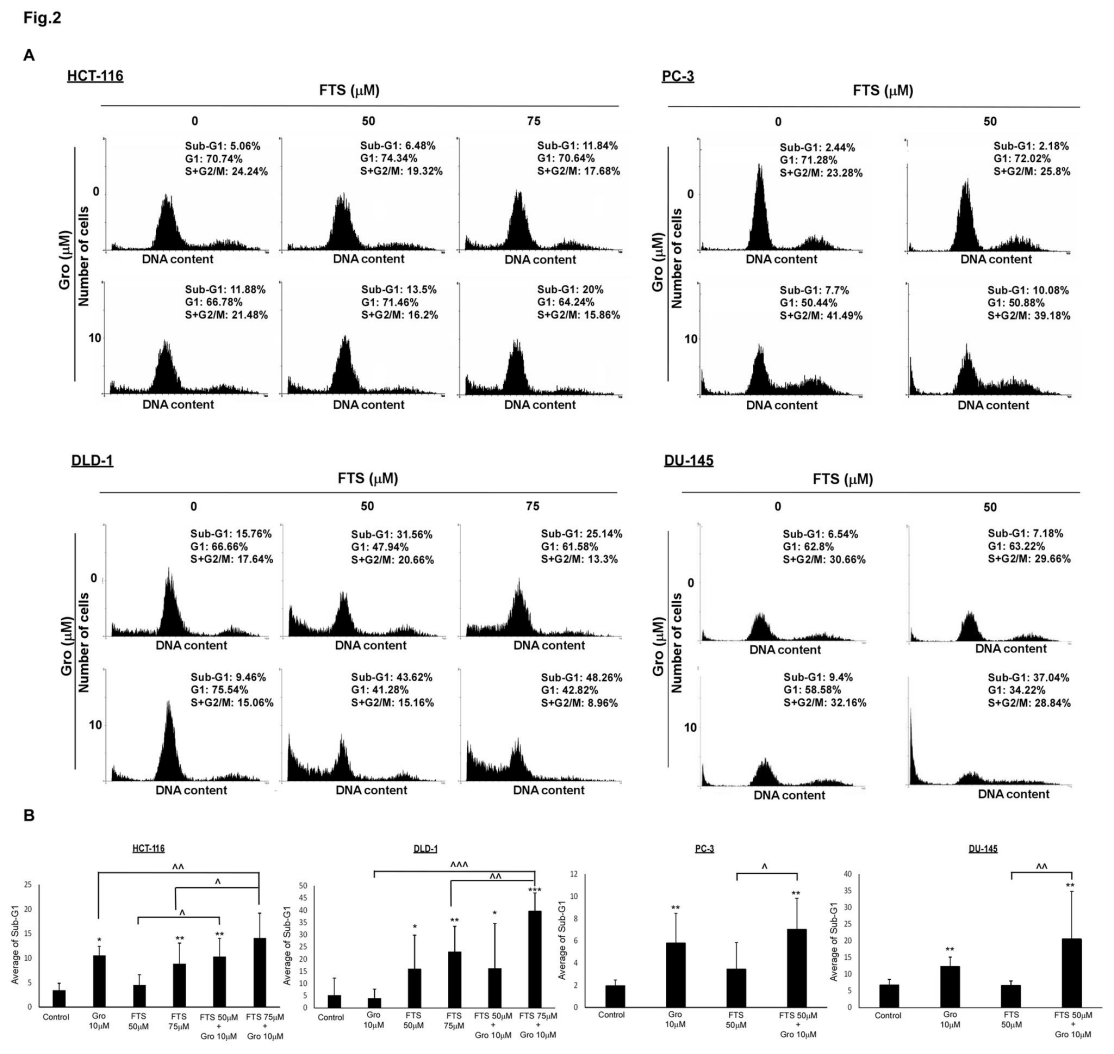
Co-treatment with FTS and GroA increases the percentage of cells in sub-G1 fraction. PC-3, DU-145, HCT-116 and DLD-1 were treated for 3 days (DU-145) 4 days (PC-3 and HCT-116) or 5 days (DLD-1) with 50 or 75 µM FTS, in the presence or absence of 10 µM Gro. The cells were then harvested and analyzed for their DNA content by flow cytometry. The percentage of live cells at different cell cycle stages is indicated. (A) Representative results of each cell line. The percentage of live cells at different cell cycle stages is indicated. (B) Statistical analysis of three typical results is presented with p-values: * ,p < 0.1 ; ** p < 0.05 ; *** p < 0.005 each treatment compared to the control ; ^ p < 0.1 ; ^ ^, p < 0.05 ; ^ ^ ^, p < 0.005 combination compared to each treatment alone.

**Figure 3:**
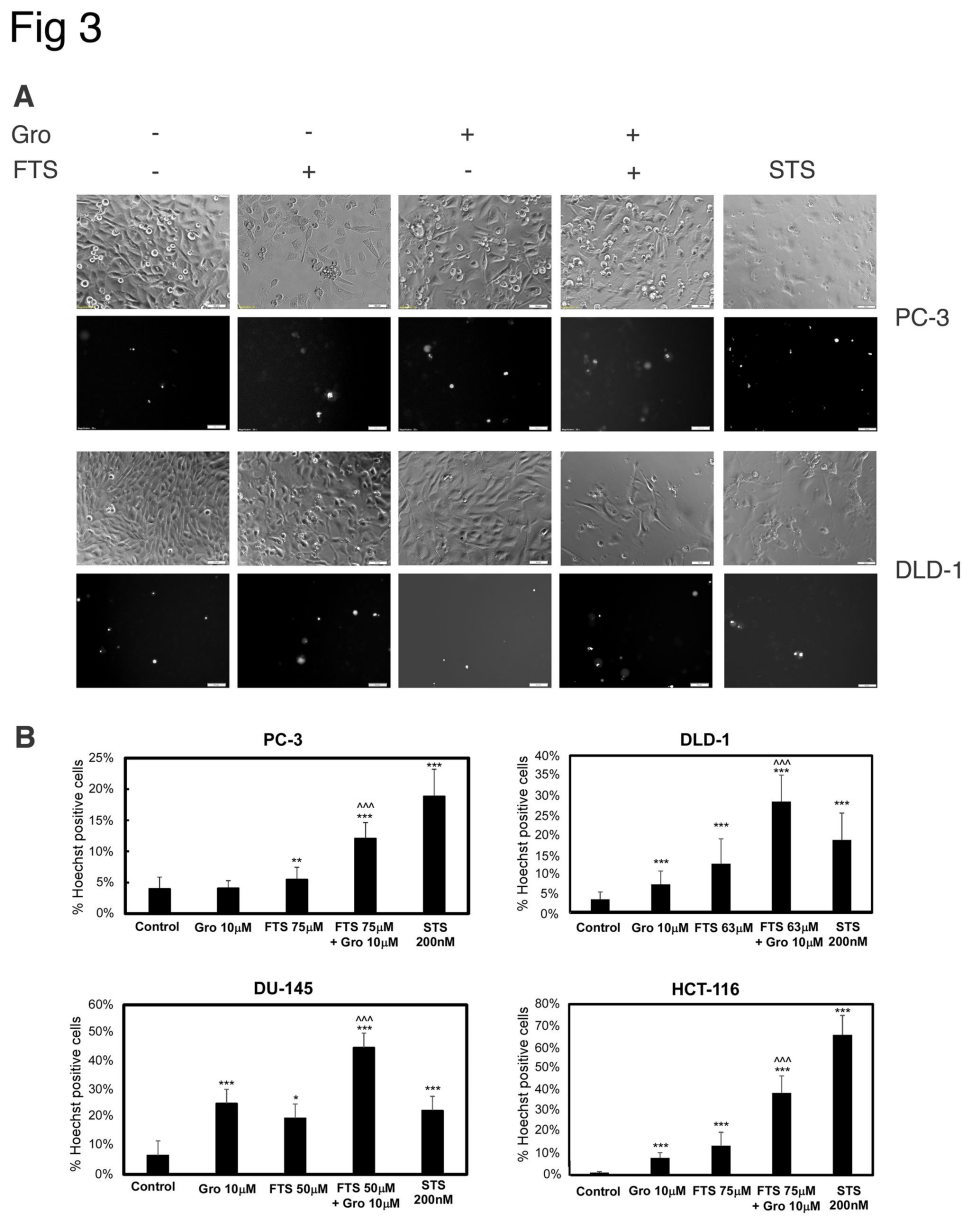
GroA enhances the inhibitory effect of FTS on cell viability. PC-3, DU-145, HCT-116 and DLD-1 cells were treated for 5 days (PC-3, DU-145 and HCT-116) or 6 days (DLD-1) with FTS (63 or 75 μM) in the presence or absence of 10 µM GroA. As positive control, the cells were treated with 200 nM STS (staurosphorine). The treated cells were then stained with the fluorescent DNA dye bisbenzimide (Hoechst 33258, 1 μg/ml) to determine the number of dead cells. After staining, the cells were photographed using Olympus motorized inverted research microscope Model IX81 (20×magnification; scale bars: 50 micrometer). (A) Representative images of PC-3 and DLD-1 cells. (B) In each field (10–15 fields for each treatment) the percentage of dying cells was estimated by counting the Hoechst-positive cells relative to the total number of cells (100–200 cells per field), and expressing the result as a percentage of the total cell number. Results are presented as mean ± S.D (*, p < 0.1 ; **, p < 0.05 ; ***, p < 0.005 each treatment compared to the control ; ^ ^ ^, p < 0.005 combination compared to each treatment alone).

**Figure 4:**
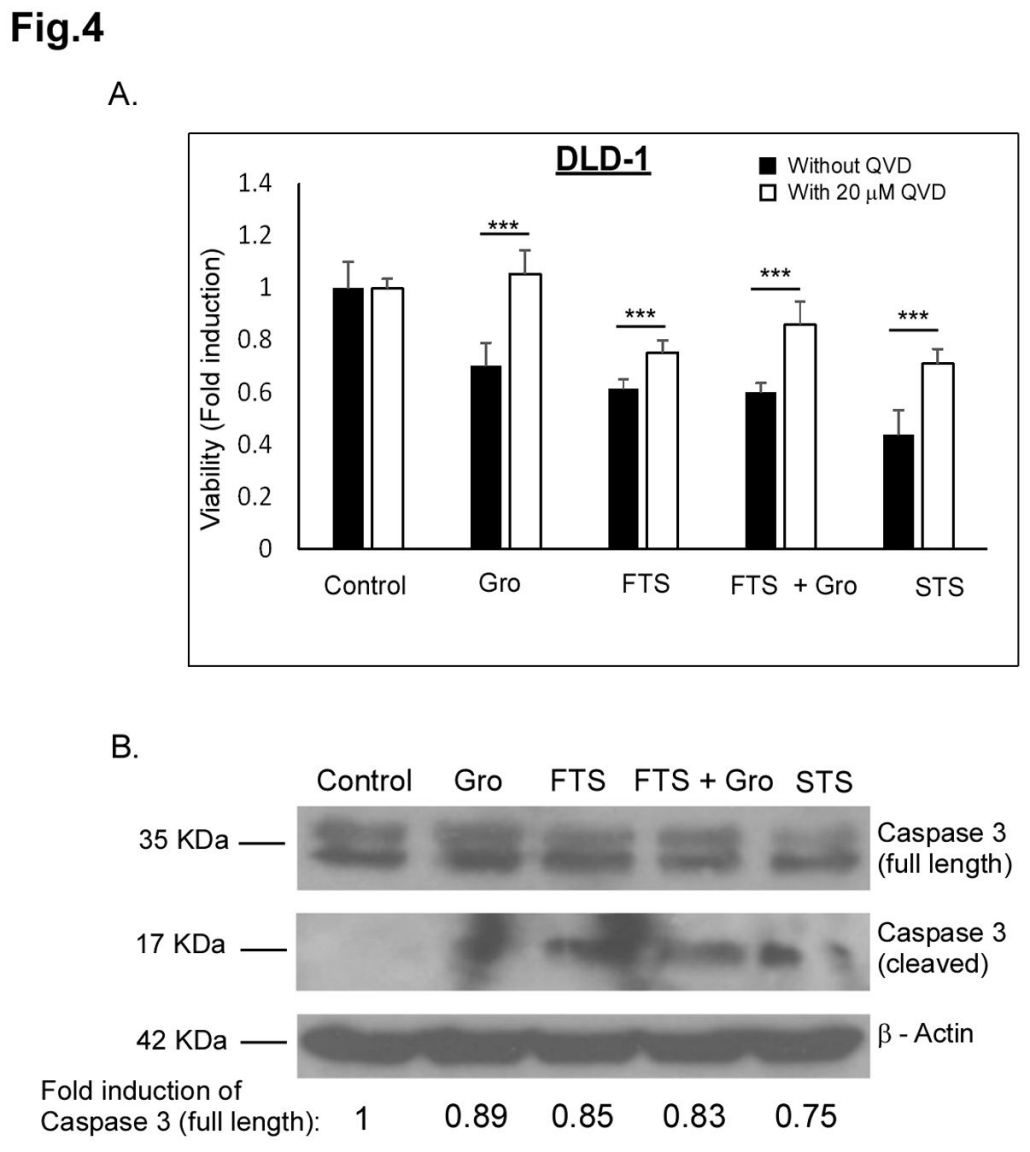
QVD rescue from GroA and FTS induced cell death. (A) DLD-1 cells were treated with 63 µM FTS with or without 10 µM GroA in the presence or absence of 20 µM QVD. The cells were then tested for cell viability using the methylene blue staining assay. (B) DLD-1 cells were treated with 63 µM FTS with or without 10 µM GroA. As positive control, the cells were treated with 200 nM STS (staurosphorine). Total cell lysates were analyzed by Western blotting, using anti-Caspase 3 Abs. As a control, total cell lysates were immunoblotted with anti-Actin Abs.

As described above, FTS and GroA co-treatment reduced the number of cells. To determine the degree of cell death induced by the combined treatment, we used two methods of cell death detection: flow cytometry and Hoechst dye exclusion assay. Cells were treated with FTS, with and without GroA for 3-5 days as indicated and then analyzed by flow cytometry. As shown in Figure 2, in all the cell lines examined, in the combined treatment, the sub-G1 population was significantly increased compared to each treatment alone. Using the Hoechst dye exclusion assay, we also observed an increase in cell death, 5-6 days following treatment with FTS combined with GroA, as indicated. As shown in Figure 3, treatment with either FTS or GroA increased cell death (presented as Hoechst positive cells). However, the combined treatment significantly increased the percentage of Hoechst-positive cells compared to each treatment alone. As a positive control for cell death induction we used staurosporine treatment, which is a known apoptosis inducer. Thus, our results strongly suggest that the combined treatment of FTS and GroA not only inhibits cell growth, but also enhances cell death. Next we examined if the cell death induced by the drugs is caspase dependent (Figure 4). DLD1 cells were treated with FTS and GroA in the presence or in the absence of broad spectrum caspase inhibitor, Q-VD(OMe)-OPh, as indicated. As shown in Figure 4A, caspase inhibition significantly inhibited FTS and GroA induced cell death. Moreover, when we examined the levels caspase 3 (Figure 4B), we could observe a decrease in full-length caspase 3 following FTS and GroA treatments. These results suggest that at least part of the cell death induced by FTS and GroA treatments is apoptotic.

### The combined treatment inhibits anchorage independent growth

**Figure 5:**
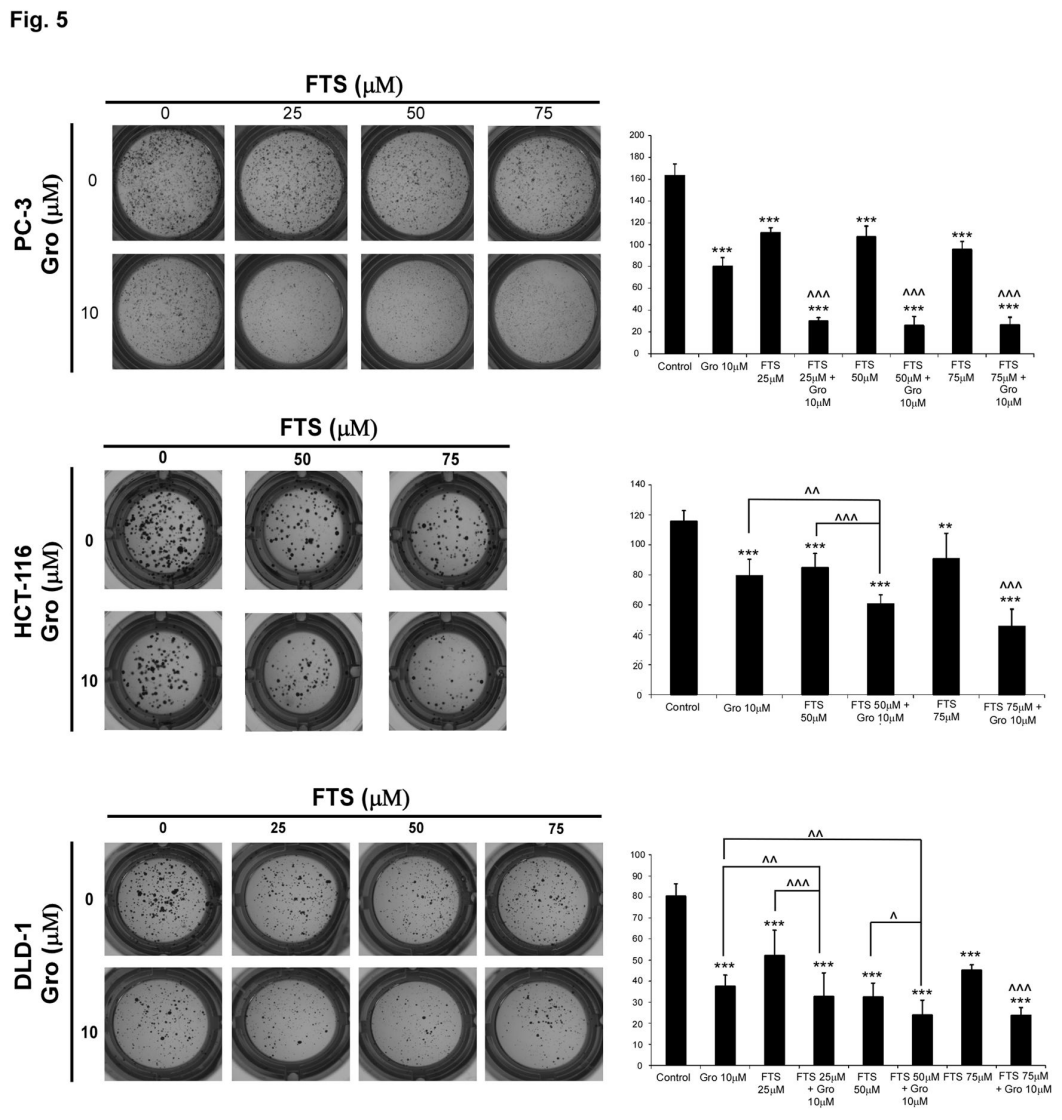
GroA enhances the inhibitory effect of FTS on anchorage independent growth. PC-3, HCT-116 and DLD-1 cells were seeded into soft agar and immediately the cells were treated with FTS (25, 50, 75 µM) in the presence or absence of 10 µM GroA. The extent of colony formation was determined 14 days (PC-3 and DLD-1) or 9 days (HCT-116) later. Colonies were then stained with MTT as described in Materials and Methods. *Left* *panels*: photomicrographs of typical wells. *Right panels*: number of colonies (>0.01 mm^2^) is presented for each treatment as the mean ± SD (n=6; * p<0.1, ** p < 0.05 and *** p < 0.005, each treatment compared to the control; ^ p<0.1, ^ ^ p < 0.05 and ^ ^ ^ p < 0.005 combination compared to each treatment alone).

We have previously demonstrated that overexpression of the three oncogenes: ErbB1, Ras and nucleolin, induced the formation of a significantly higher number of large colonies in soft agar as compared to clones expressing only two of the proteins [27]. Thus, we next examined whether the combined treatment with FTS and GroA will affect anchorage independent growth. For this aim, we employed the soft agar assay. Cells were plated in soft agar and maintained in culture for 9-14 days, as indicated, before quantifying the number and size of colonies able to grow in an anchorage-independent manner. Results of typical experiments are shown in Figure 5. Under the conditions tested, the combined treatment induced a significantly lower number of colonies in all three cell lines tested. Thus, our results indicate that treatment with FTS and GroA inhibits cell transformation.

### The effect of FTS and GroA on cell migration

**Figure 6:**
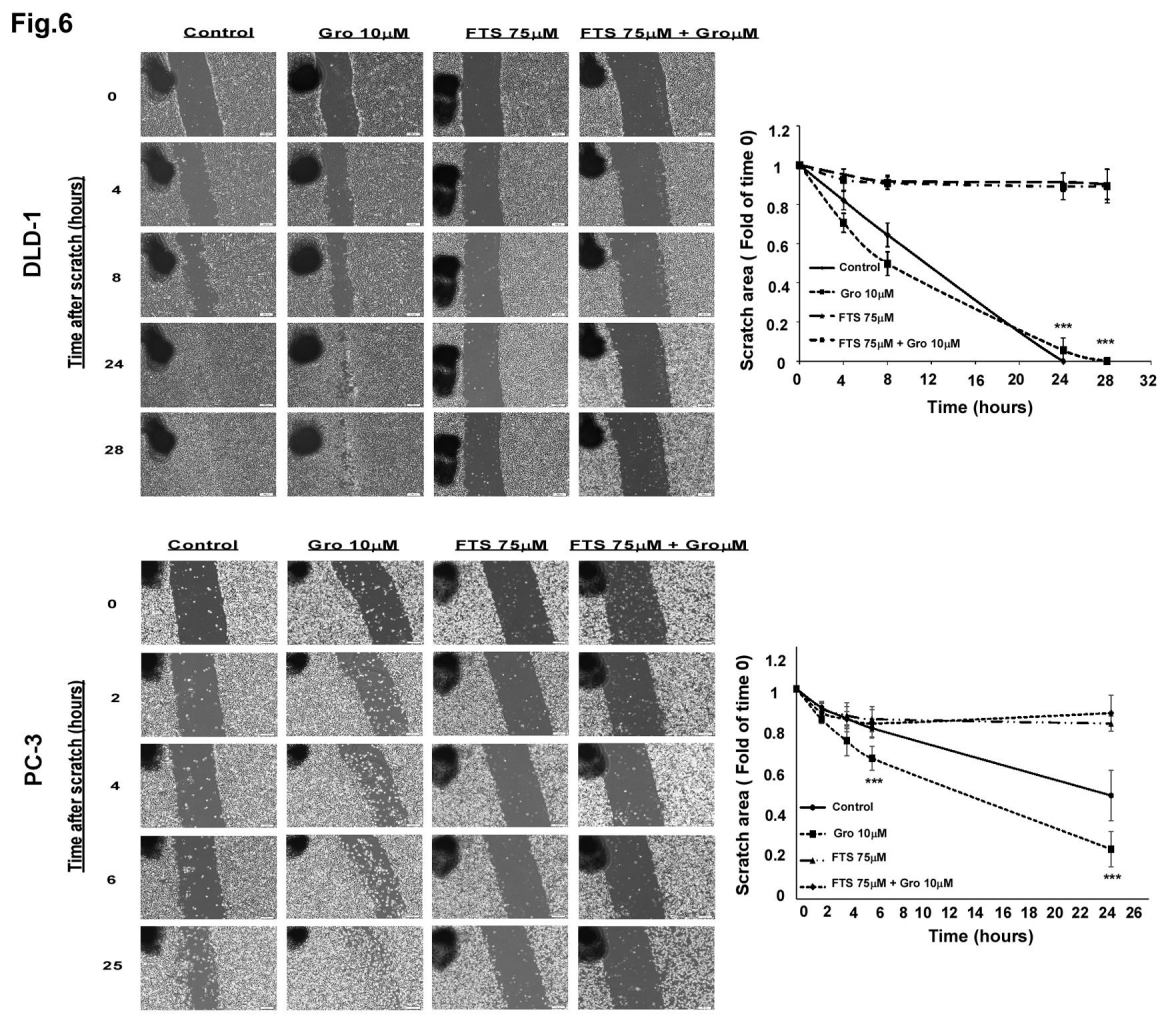
Co-treatment of FTS and GroA affects cell migration. DLD-1 and PC-3 cells were treated with 75 µM FTS in the presence or absence of 10 µM GroA. A day later, a scratch wound was inflicted at each well and the resulting gap was imaged at the indicated time points. *Left* *panels*: representative images (4xmagnification; scale bars, 200 micrometer). *Right panel*s: the width of the scratch was quantified using ImageJ; the results are presented as fold over time 0 and are mean ± S.D (*, p < 0.1; **, p < 0.05; ***, p < 0.005). The experiments were performed in triplicates and repeated twice.

In order to study cell motility, scratch-induced migration assay was employed [35,36]. The cells were treated for 24 hours with and without FTS, in the presence or in the absence of GroA. In each well, three areas were scratched, creating three gaps of similar widths. The media and the inhibitors were then replenished. Immediately thereafter, and at the indicated time periods, phase-contrast images of the plates were obtained. The widths of gaps following treatment with the different inhibitors and at different time points were measured as described in the methods section. Results of typical experiments are presented in Figure 6. As shown, the gap closure was inhibited by FTS treatment but not by GroA treatment. However, in the combined treatment GroA did not interfere with the effect of FTS on the cells’ motility and the gap remained unclosed. Thus, we concluded that FTS inhibits cell migration, which is not affected by GroA treatment.

### FTS and GroA effect on Ras and nucleolin localization

**Figure 7:**
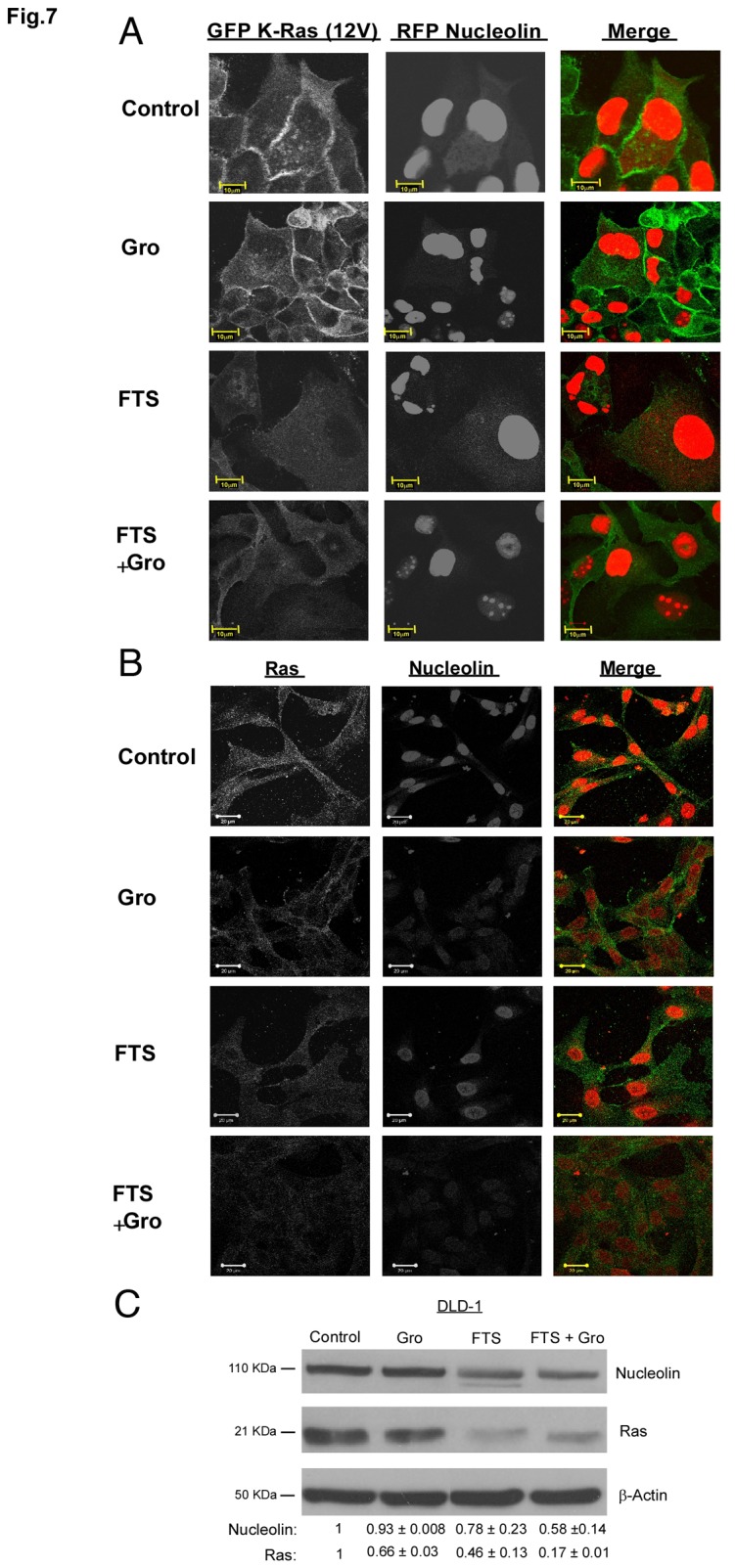
FTS and GroA treatment affect Ras and Nucleolin localization and reduce their levels. MDCK and DLD-1 cells were seeded on coverslip coated with poly-L-Lysine and treated with 63 µM FTS (DLD-1) or 75 µM FTS (MDCK) in the presence or absence of 10 µM GroA. After 3 days, cells were fixed and examined by confocal microscopy at 63×magnification using Zeiss 510 META confocal microscope. (A) Representative images of MDCK cells following treatments. (B) Representative images of DLD-1 cells following treatments. (C) DLD-1 cells were seeded at a density of 1.5×10^5^ cells per well in six-well plate in RPMI medium supplemented with 5% FBS. A day later, the cells were treated with 63 μM FTS in the presence or in the absence of 10 μM Gro for 3 days. Total cell lysates were analyzed by Western blotting, using anti-nucleolin and anti- pan Ras Abs. As a control, total cell lysates were immunoblotted with anti-β Actin Abs. Note that GRO and FTS treatments reduced the nucleolin and Ras levels respectively in response to each treatment, however the combined treatment of FTS and GroA reduced the nucleolin and Ras levels simultaneously.

As described previously, there is a crosstalk between the three proteins: ErbB1, Ras and nucleolin [25-27]. Previous experiments have shown a co-localization of the three proteins on the plasma membrane [27]. Hence, we examined whether co-treatment with FTS and GroA will affect the co-localization of the proteins on the plasma membrane. For this aim, we used MDCK cells stably expressing ErbB1, GFP-K-Ras (12V) and RFP-Nucleolin. The cells were treated with FTS (75 µM), GroA (10 µM) or both for 3 days. As shown in Figure 7A, in the control, the K-Ras protein (in green) is mainly localized on the plasma membrane. Nucleolin (in red) is localized mainly in the nucleolus but also at the cytoplasm and on the plasma membrane. When cells were treated with GroA, the total amount of nucleolin was reduced, especially from the plasma membrane and cytoplasm, but there was no significant change in the localization of the K-Ras protein. When cells were treated with FTS, K-Ras dislocated from the plasma membrane, but there was no effect on the intracellular localization of nucleolin. However, the combined treatment affected both protein levels and localization. The two proteins were dislocated from the plasma membrane and their levels were reduced. Next we examined whether the colocalization is also affected in DLD-1 cells that endogenously express the proteins. As shown, FTS and Gro treatments affected the levels and the localization of Ras and Nucleolin (Figure 7B). To further support these results, we examined the effect of the treatments on nucleolin and Ras levels by immunoblot analysis. As shown in Figure 7C, the combined treatment reduced the levels of Ras and nucleolin in DLD-1 cells. Taken together, our results suggest that the combined treatment reduce the levels of Ras and nucleolin.

## Discussion

In our previous reports, we have demonstrated that nucleolin interacts with ErbB receptors and Ras proteins. This interaction leads to enhanced cell growth and cell transformation [25-27]. Since the three oncogenes interact and this interaction increases their oncogenic potential, we have set up experiments to test the effect of treatments with drugs that inhibit two of the interacting proteins; FTS that inhibits Ras and GroA that inhibits nucleolin. Thus, we examined whether treatment with FTS and GroA will inhibit cell growth and cell transformation. Our study focused on two colon cancer cell lines, that harbor K-Ras mutation, and two prostate cancer cell lines, that express high levels of ErbB receptors. In these four cancer cell lines our results demonstrate that the combined treatment of FTS and GroA reduces cell transformation more effectively than treatment with each drug alone. These results are important in light of the drug resistance observed in colon cancers that express K-Ras mutation and do not respond to anti-ErbB drug treatments [37].

Previously, it was demonstrated that Ras inhibition by FTS could inhibit the anchorage-independent growth of various cancer cells [11,12]. Furthermore, FTS inhibits growth and can induce apoptosis of cancer cell lines such as hepatocarcinoma and prostate cancer [12,38]. Nevertheless, in a large number of cancers, tumor cells do not undergo apoptosis when treated with FTS. These include pancreatic [13], colon [14] and lung cancer cell lines that express mutant K-Ras [15]. Our results show that FTS treatment with or without combination with GroA, not only induces cell growth inhibition, but can also significantly enhance cell death induced by each of the drugs alone. The cell death induced by the drugs may be partially apoptotic since it is inhibited by the broad-spectrum caspase inhibitor.

Tumor metastasis is a leading cause of cancer patients’ death. Cell migration is a critical step in tumor invasion and metastasis. Therefore, regulation of this process is highly important [39-41]. In the present study, we found that cell motility was inhibited by FTS and not by GroA treatment. In the combined treatment, however, GroA did not interfere with the effect of FTS on cell motility inhibition. These results suggest that both drugs may affect different cellular pathways, as they induce different biological responses.

In our earlier studies, we demonstrated co localization of nucleolin, ErbB1 and Ras on the cell’s plasma membrane [25,27,42]. We also demonstrated an oncogenic cooperation between these proteins, suggesting that targeting these proteins may yield better response. Indeed, our results demonstrated that the combined treatment with both inhibitors affected Ras and nucleolin localization. In conclusion, our results indicate that treatment with a combination of inhibitors that target the oncogenic cooperation between ErbB1, Ras and nucleolin, has the potential to better inhibit tumor cell growth. In addition, the combined treatment increases cell death, inhibits cell transformation, inhibits cell migration and affects protein localization. These results, showing a better effect of the combined treatment compared to treatment with each of the drugs alone, may explain the drug synergism we observed. Furthermore, our study suggests a new and possibly more effective way to treat cancer patients who harbor overexpression of these oncogenes.
